# Migration crisis in the EU: developing a framework for analysis of national security and defence strategies

**DOI:** 10.1186/s40878-018-0093-3

**Published:** 2018-10-01

**Authors:** João Estevens

**Affiliations:** Portuguese Institute of International Relations, Nova University of Lisbon, Lisbon, Portugal

**Keywords:** Migration-security nexus, Migration crisis, Security and defence strategies, Comparative research, EUGS

## Abstract

This article addresses migration-security nexus in the EU by assessing Member States’ national security and defence strategies as well as the 2016 EUGS in a time of migration crisis, a crisis that stands as one of the most important geopolitical challenges today in the EU. After developing and applying a framework for analysis derived from a literature review, the existing differences among Member States are clear in terms of strategic cultures and approaches to migration issues. The idea of ‘EU’rope without internal borders is at stake as Schengen is under serious attack due to increasing Eurocentrism and growing extreme right-wing populism, which are a consequence of increasing economic protectionism and international terrorism. The solution seems to depend on two critical uncertainties: the evolution of political and social instability in the North Africa and the Middle East, and the future of the EU itself. The results enlighten a securitization of migration mostly centred on the nation-state and national security rather than on people and human security.

## Introduction

The twenty-first century is the century of the migrant, being global mobility a highly stratified phenomenon, from the global tourist to the undocumented employee, and from human trafficking to refugees forced to leave their country of origin because of climate changes, poverty or wars (Castles & Miller, 2009). Hence, (forced) migration is contributing to changes in structures and institutions in global political, economic and social relationships (Castles, 2010, p. 1566). Nowadays, there are two large demographic trends in the European Union (hereinafter EU): continuous population ageing and increasing migration flows (European Commission, 2014), both relevant to study the relation between demography and security though this article only focuses on the second one. The abolition of internal borders within the EU fostered the dissemination of narratives that suggest a security deficit and new challenges to public order derived from the opening of internal borders, leading to increasing politicization and securitization of migration and asylum issues (Huysmans, 2006; Guild, 2009; Bourbeau, 2011; Vietti & Scribner, 2013). This de-nationalization of state sovereignty demands cooperation and though close cooperation in security and defence appears to be indispensable in the EU, there is no common ‘EU’ropean position on how to deal and how to move beyond the humanitarian crisis management after the so-called refugee crisis, which can rely on diverging national strategic cultures that end up enforcing different security and defence policies within the EU (Biehl, Giegerich, & Jonas, 2013). When studying migration, terminologies matter. One important term is refugee, which refers to a political-legal status under the 1951 Refugee Convention and its 1967 Protocol framed within international law. Most of the recent EU institutional documents make that distinction, since many of the forced migrants are still waiting for their legal status to be defined, being therefore not yet named refugees, but asylum-seekers due to several bureaucratic constraints (Bello, 2017, pp. 55–56). So, I will avoid using the expression ‘refugee crisis’ and will instead use ‘migration crisis’, considering in here different categories of forced migrants, both asylum-seekers and refugees.

In this article, I aim to explore how the EU and Member States (hereinafter MS), actors influenced by NATO security strategy, approach migration-security nexus in a time when forced migration still stands on top of the EU’s policy agenda, especially in the context of the ongoing Syrian civil war and with several fragile States like Iraq, Afghanistan, Libya or Egypt, among others, in the MENA region. The EU has been facing a record numbers of migrants, asylum-seekers and refugees in recent history that ignited stronger border control and several difficulties in the management of these flows, expressed through mass detention of new arrivals, lack of organization and resources in refugee camps, dual negotiations with transit countries, increasing human trafficking networks and the lack of solidarity and agreement about the European relocation scheme.

So, the questions addressed by this article are, first, what are the key issues that compose the migration-security nexus? Following from this, the article examines the presence of those key issues institutionally, both in national security and defence strategies and in the EU Global Strategy (hereinafter EUGS), to have an overview of differences among MS and the EU in the securitization of migration. I address the relationship between security and migration through a comprehensive approach to the existing literature and focus on MS security and defence strategies (White Papers, Defence Agreements, Defence Concepts or another similar strategic document), except for Cyprus, whose strategy wasn’t available to download. The period considered for the collection of the documents varied because I used the most recent document per MS regardless the date of release. Laying on the assumption that security has a political nature (Huysmans, 2006, p. 145) and assuming security as a continuum involving various degrees of intensity (Bourbeau, 2011, p. 18), these documents are key instruments to understand if migration is perceived as a relevant security matter that requires political action, and so essential to analyse the strategic securitization of migration and its intensity (Lazaridis, 2015, p. 108).

After applying a framework for analysis derived from a literature review, I argue that there is a heterogeneous presence of migration issues among national security and defence strategies of MS due to different security strategic cultures and approaches to migration-security nexus, which block the development of a common and effective strategy to deal with the recent migration crisis. Though 2016 EUGS enlightens this crisis and tries to get some common ground among MS, there are many issues that remain underexplored and so the ‘EU’ropean political action is still an undergoing process. That reflects a lack consensus among MS and the strong intergovernmentalism in the EU about security matters, being it one of the key fields where MS cooperate in specific areas while retaining their sovereignty in others. So, all MS tend to impose their own domestic protection standards about EU asylum policies to the EU level, but some MS are more effective influencing EU policies than others, therefore existing stronger regulating MS impacting on EU migration and asylum legislative outputs (Zaun, 2017, pp. 13–14). Lastly, I also conclude that the securitization of migration seems to be much more focused on the nation-state rather than on people and so a national security approach is still prevailing over a human security approach.

## Migration-security nexus

Security is built on a set of discourses or narratives and historical practices based on institutionally shared understandings, therefore becoming a political and social construct (Wæver, 1995). During this process, the elites in power, analysts and experts define the existing risks and threats in a certain moment and for different levels (national, regional, global). Then, they justify their validity alongside the community, subsequently activating, when possible, the means to neutralise them. Thus, the inclusion of a specific approach to security, in state practices or in international organisations, tend to be derived from an existing structure of power. The process of globalisation has added new functions to state responsibility and changed some of the previous ones, since the traditional function to guarantee the defence of its territory and political independence is now attached to the obligation of assuring economic independence, cultural identity and social stability. Globalisation has been transforming the existing risks and threats, which are impossible to neutralise by only focusing on the state and with a national security strategy limited to national boundaries (Sørensen, 2005; Mabee, 2009; Ripsman & Paul, 2010).

During the nineties, after the peaceful ending of the Cold War, the growth in intra-state conflicts, Western societies’ fear of immigration, the decaying environment and the acceleration of the HIV/AIDS epidemic, it was inevitable to include new strategic factors associated with human security. The acknowledgement of a new world led to new developments on security, from its traditional political-military conception centred in the state and its sovereignty, to a more inclusive and holistic view of peace and international stability based on the protection of individuals (Buzan & Hansen, 2009, p. 187). The state stopped being the only referent object for security. Nonetheless, human security did not replace national security, integrating new dimensions as the protection of human rights, economic development and individual security. It had an institutional conceptualization in the UN 1994 *Human Development Report* and was characterized by a universal, broad and flexible approach and by the interdependencies among the seven components: economic security, food security, health security, environmental security, personal security, community security and political security (UNDP, 1994). The broadening of the security concept was pushed by the Copenhagen School, a school of academic thought on security studies that emphasized social dimensions of security and rejected the sovereign state as the primary referent for and agent of security, also defending increasing difficulties to sustain an enduring and reliable national security strategy without a strong response to human insecurity (Vietti & Scribner, 2013, p. 27). It has also implied the assumption of ‘new’ risks and threats that demand new processes of securitization, spurring preventive diplomacy, good governance and economic and social development to save a society from reaching a crisis point. Population dynamics have become more important with the rise of human security as it represents a major source of pressure for the security of individuals and, consequently, for national security (Tragaki, 2007; Vietti & Scribner, 2013).

The interest in how population studies and security studies are connected is increasing very fast in the last decades, drawing on a rich literature on this subject (Weiner & Russell, 2001; Bigo, 2002; Goldstone, 2002; Guild & van Selm, 2005; Adamson, 2006; Huysmans, 2006; Bourbeau, 2011; Sciubba, 2011; Goldstone, Kaufmann, & Toft, 2012; Rodrigues, 2015). Also, demography-related risks can produce feedback effects due to strong interdependence among risks and threats, and so demography matters for national and global security in different ways (Urdal, 2005; Black et al., 2011; Goldstone, Kaufmann, & Toft, 2012; Rodrigues, 2015). Migration can matter for national security in situations when migrants or refugees are opposed to home countries’ regime, when they are perceived as a security risk or a cultural threat in the home country, when immigrants cause social and economic pressure in host societies, or when the host society use immigrants as an instrument against the country of origin (Weiner, 1992, pp. 105–106). The securitization of migration tends to include four different axis: socioeconomic, due to unemployment, the rise of informal economy, welfare state crisis, and urban environment deterioration; securitarian, considering the loss of a control narrative that associates sovereignty, borders, and both internal and external security; identitarian, where migrants are considered as being a threat to the host societies’ national identity and demographic equilibrium; and political, as a result of anti-immigrant, racist, and xenophobic discourses (Ceyhan & Tsoukala 2002, p. 24). Therefore, since migration can impact in different areas as state sovereignty, the balance of power among states and the nature of conflicts in the international system, national security may also be affected (Adamson, 2006). Lastly, increasing human mobility has been associated with: urban clusters for migrants (Rodrigues, 2015, pp. 45–46), the capacity of states to control entry, primarily in terms of illegal entry (Mabee, 2009, pp. 123–124), and on asymmetries in ethnic and religious population composition (Tragaki, 2007, p. 105). If immigrants are not integrated into host communities, particularly if they come from a completely different cultural environment, the potential risk of religious and ethnic conflicts tends to be higher, demanding new governmental integration efforts of ethnic minorities into national communities (Savage, 2004).

It is also necessary to weigh up the security of immigrants themselves, especially when they try to enter the host country illegally because of human trafficking networks (Czaika & de Haas, 2013), that have caused the loss of human lives, notably in routes from North Africa to Southern European countries and, more recently, related to the Syrian civil war (Ferreira, 2016a, pp. 1–2). Nevertheless, even if they settle in, some reports indicate cases of illegal work, work exploitation, involvement in prostitution and human organ trafficking networks (Burgess, 2011, p. 15), which generate a space for the legal marginalisation of (im)migrants based on the of use nationalistic values to justify the exclusion of immigrants (Geddes, 2003, p. 22). For the benefit of immigrants and hosting communities, prevention of marginalisation, discrimination, urban segregation and social disruption are essential to ensure social stability. If economic and social security of these individuals is granted, the area of socio-economic exclusion will be restricted. However, so far, the path followed does not seem very inclusive, as some countries in global North rely on growing punitive governance, which clearly illustrates the cycle of labour marginalisation, radicalisation and criminalisation. The politics of immigration control and the criminalization of illegal immigrants derive mostly from a social-legal system that allow their marginalization and their labour exploitation (Melossi, 2015, p. 60, p. 85).

## Different approaches to migration-security nexus among member states

It is impossible to ignore the impact of international terrorism in the development of migration-security nexus. The current moment of terrorism inside the EU, especially after recent attacks, may result in a similar situation as has occurred in the USA after 9/11, with the inclusion of immigration issues in the anti-terrorism agenda. Indeed, it was one of the reasons why migration has become an intense object for security analysis (Burgess, 2011, p. 14). Terrorism shapes public opinion on migration, which justifies the need for better understanding about terrorism and religion. Failing this, one may assume that any Muslim may be potential terrorist, which might give rise to Islamophobia inside Europe, as data seem to support that Muslims suffer from higher levels of discrimination in Western democracies, in comparison with other religious minorities, especially since 2001 (Fox & Akbaba, 2015, p. 191). Political action and the media are key elements to deconstruct these associations between immigration and terrorism, and between immigration and criminality, thus encouraging more tolerance and acceptance. The securitization of immigration is both an outcome and a cause of extremist narratives inside right-wing political parties (Bigo, 2002, p. 65). The increase in immigration flows should reinforce integration policies and narratives to avoid the rise of right-wing populism in response to unbalanced immigration within extreme right-wing and Eurosceptic parties that find in the post economic crisis context new discontent electorate willing to support a conservative approach to migration issues (Vieten & Poynting, 2016; Liang, 2016). Also, the rise of extreme right-wing parties with populist narratives may encourage the development of social movements with radical factions that may be more eager to carry out terrorist attacks or participate in jihadist recruiting, finding their legitimacy inside the social movements springing from this change in the MS political systems.

Historically, most nation-states define themselves in ethnic rather than civic terms, allowing little room for ethnic and cultural diversity (Lazaridis, 2015, p. 142). Nowadays, identities may not correspond to the borders of national sovereignty, triggering a reconceptualization of the traditional national identity associated with nationality. Also, by taking into consideration the data on foreign population (%) and gross emigration/immigration rates, it is possible to conclude a dissimilar dependence on migration among MS (European Commission, 2014). That makes relevant to analyse the current levels of ethnic-religious fragmentation in EU Member States. Higher levels of ethnic, religious or cultural fragmentation seem evident in Eastern European countries, but they are also present in Western Europe in countries like Belgium, France, Germany, Luxembourg, Spain or the United Kingdom (Alesina, Devleeschauwer, Easterly, Kurlat, & Wacziarg, 2003; Fearon, 2003; Patsiurko, Campbell, & Hall, 2012).

### National security and defence strategies

The evaluation of national strategies is based on similar strategic documents that are essential to understand MS strategic cultures and institutional securitization of migration, though they can present some variation due to overall differences in content and form, particularly in: (i) defining the security concept approach, (ii) characterising the national and global security environment, (iii) integrating the demography into the strategy. Since there is not a unitary definition of what strategic culture is among scholars, I follow Biehl, Giegerich, & Jonas (2013, p. 11) view on this, understanding “*strategic culture as a variable that structures what options are considered to be appropriate by a specific actor in security and defence, hence influencing, but not determining, behaviour*”. Having these assumptions in mind, all documents reflect each Member State decision-makers and experts’ preferences in security and defence policy at a specific moment in time about different issues, being migration one of those. These documents were planned and implemented at different times and try to give a persistent security strategy projection as they are expected to last four, five, ten or fifteen years, except for Luxembourg (one year). This justifies the exclusion of conjunctural dynamics because the structural ones supersede temporary security drivers. As shown in Table 1, the level of exploration of migration-security nexus, according to a content analysis, seem to differ both in terms of extension, number of references and issues approached in MS national security and defence strategies.

**Table 1:** Migration-security nexus in national security and defence strategies

Country		Document year	None or low exploration	Medium exploration	High exploration
Austria	(Federal Ministry Defence Austria, 2012)	Weißbuch des Bundesheeres (2012)	✓		
Belgium	(Ministery of Defense Belgium, 2016)	La vision stratégique pour la Défense (2016)		✓	
Bulgaria	(Ministry of Defence of the Republic of Bulgaria, 2010)	White Paper on Defence and the Armed Forces of the Republic of Bulgaria (2010)	✓		
Croatia	(Ministry of Defence Republic of Croatia, 2013)	Strategic Defence Review (2013)	✓		
Czech Republic	(Ministry of Foreign Affairs of the Czech Republic, 2015)	Security Strategy of the Czech Republic (2015)			✓
Denmark	(Danish Ministry of Defence, 2012)	Danish Defence Agreement 2013-2017 (2012)	✓		
Estonia	(Estonian Ministry of Defence 2011)	National Defence Strategy Estonia (2011)	✓		
Finland	(The Government of Finland, 2012)	Finnish Security and Defence Policy (2012)			✓
France	(Ministry of Defence France, 2013)	French White Paper. Defence and National Security (2013)	✓		
Germany	(German Ministry, 2016)	White Paper on German Security Policy and the future of the Bundeswehr (2016)			✓
Greece	(Ministry of National Defence, Greece. 2014)	White Paper on Defence (2014)			✓
Hungary	(Ministry of Foreign Affairs of Hungary, 2012)	Hungary’s National Security Strategy (2012)			✓
Ireland	(Department of Defence Ireland, 2015 )	White Paper on Defence (2015)		✓	
Italy	(Ministery of Defense Italy, 2015)	White Paper for International Security and Defence (2015)		✓	
Latvia	(Ministry of Defence of the Republic of Latvia, 2012)	State Defence Concept (2012)	✓		
Lithuania	(Ministry of National Defence Republic of Lithuania, 2017)	National Security Strategy (2017)			✓
Luxembourg	(The Luxembourg Government, 2016)	Déclaration de politique européenne et étrangère (2016)		✓	
Malta	(The Armed Forces of Malta, 2010)	The Armed Forces of Malta and Military Doctrine (2010)	✓		
Netherlands	The Dutch Defence Doctrine (Ministry of Defence of the Netherlands, 2013)	Netherlands Defence Doctrine (2013)		✓	
Poland	(National Security Bureau, 2013)	White Book on National Security of the Republic of Poland (2013)			✓
Portugal	(Republica Portuguesa, 2013)	Conceito Estratégico de Defesa Nacional (2013)		✓	
Romania	White Paper on Defense Romania (Ministry of National Defence of Romania, 2015)	White Paper on Defense (2015)	✓		
Slovakia	(Ministry of Defence of the Slovak Republic, 2013)	White Paper on Defence of the Slovak Republic (2013)		✓	
Slovenia	(Republic of Slovenia, 2010)	Resolution on National Security Strategy of the Republic of Slovenia (2010)			✓
Spain	(Government of Spain, 2013)	Estrategia de Seguridad Nacional: un proyecto compartido (2013)			✓
Sweden	A Functional Defence. A summary of the Government Bill (Government Offices of Sweden, 2015)	Functional Defence: Government Bill (2015)	✓		
United Kingdom	(Ministry of Defence, UK, 2015)	National Security Strategy and Strategic Defence and Security Review 2015 (2015)			✓

A higher presence could be inferred from the most recent strategies following the 2015 mediatized migration crisis (Belgium, Czech Republic, Germany, Ireland, Italy, Lithuania, Romania, Sweden and United Kingdom). However, that is unclear as there is a wider exploration of migration issues in Czech, German, Irish, Lithuanian and British documents and a weaker exploration in Belgian, Italian, Rumanian and Swedish documents. There are MS as Austria, Bulgaria, Croatia, Denmark, Estonia, France, Latvia, Malta and Sweden that give less to none importance to migration issues in their national security and defence strategies, which lead to the conclusion that migration is probably less securitized in these countries. And even the countries that explore the migration-security nexus in depth also do it in a very distinct way, tackling different issues, as one may notice below.

#### Belgium (2016)

While exploring Southern Mediterranean neighbourhood, political instability and failed States in North Africa, Sahel, Horn of Africa and Middle East are perceived as leading to irregular migration and refugees’ flows. Cooperation with the EU is essential in the stabilization of these countries and that is crucial to cope with the refugees.

#### Czech Republic (2015)

One of the most important trends and factors in the security environment is the importance of non-military threats, such as migration, placing demands on EU ability to respond independently and efficiently. Mass uncontrollable migration will come from political instability, poverty, climate change and needs to be addressed with population ageing. There are negative aspects of international migration that can lead to security threats, especially due to insufficient integration of legal migrants that can also give rise to social tensions, resulting in undesirable radicalisation of people belonging to immigrant communities. The Czech Republic applies a conceptual approach to integration of immigrants involving a broad range of actors, including non-governmental organisations and continues to encourage the merging of immigrant communities with the majority population, and the social and economic self-sufficiency of individual immigrants.

To promote the security interests of the Czech Republic is necessary to address EU and Schengen membership. Abolition of internal border control within the EU has considerable implications on the way the Czech Republic protects its territory and fights irregular migration. Cooperation among MS is needed and necessary to protect EU’s external borders. The Czech Republic advocates the completion and use of modern high-capacity information systems and the introduction of entry and exit registration systems that can contribute to the security of the common area, continuing to advocate the retention and effective administration of key EU migration policy mechanisms, including asylum policy cooperation under the Dublin System, and consistent adherence to the Schengen.

#### Finland (2012)

The security environment takes the EU into consideration as it is a major actor in many key areas related to societal development and comprehensive security. The CFSP is important for border management and immigration policies. Migration issues are particularly explored when addressing climate change and its impact on security as growing uncontrollable migrations, both inside and between states, is expected from areas that are the worst affected by climate change. The development of international cooperation is essential regarding all transnational threats. Finland will act through the EU and bilaterally to respond to border management and immigration control challenges. Finland will also participate in the development of the EU border, maritime, immigration and common asylum policies, and in the operation of the European border management agency FRONTEX; and the European Asylum Support Office.

On a side note, a reflection about Russian connection between immigration and growing nationalism is also pointed out. While emigration is on the rise, immigration is also increasing in a situation in which Russia has already received more immigrants than any other country in Europe. Immigration is one of the underlying causes for the growing nationalism in Russia.

#### Germany (2016)

Uncontrolled and irregular migration are explored as autonomous section of Challenges for German Security Policy. But first, the need for qualified immigration is addressed as there are demographic changes that cannot be stopped. To remain globally competitive, ageing societies as the German one must find new ways of receiving sufficient qualified immigrants and retaining skilled workers. Migration is also pointed out as one of the outcomes of climate change and one of the challenges that the EU and its MS are facing today.

In its own section, the drivers of migration are detailed as it is the exploitation that comes from organized crime and terrorist networks whether in countries of origin or transit ones. The economic and social gap between Europe and its neighbouring regions and the ongoing violent conflicts in many parts of the world will lead to potential migration in the coming decades. Migration does not pose a risk to Germany’s security. In large numbers, uncontrolled and irregular migration can, however, entail risks. The ability to absorb and integrate migrants can be overstretched, which can lead to social instability. Refugee movements resulting from violent conflicts can also cause such conflicts to spread throughout a region.

The causes of irregular migration must be addressed in a joint effort by the international community and the countries of origin and transit, demanding an effective European strategy and practice. It is important to provide support for internally displaced people and refugees, and Germany embraces its responsibility for managing the humanitarian consequences of refugee movements. This cooperation will lead to effective protection of Europe’s external borders. Regarding refugee and migration policies, it is important to ensure equitable burden-sharing at European level and at the same time to develop viable solutions through dialogue with the countries of origin, the initial host countries, and the transit countries.

#### Greece (2014)

The generalised instability in the Middle East, Maghreb and Eastern Mediterranean, puts Greece in an extremely exposed position to the waves of immigrants and refugees, especially taking into consideration its extended sea borders and numerous islands, islets and rock-islets that may facilitate the conduct of illegal activities. One of those is international terrorism and another one is organised crime, especially linked with a tendency of illicit human trafficking that affects mostly Southern European countries, having those to bear most of the burden of managing illegal immigrants at the European level. After, the document mentions that fact in conjunction with the distortions brought by the EU immigration policy, pose a major challenge for national and European security.

According to the Greek White Paper, illegal immigration is one of the main threats undermining national and international security. The commitment with the EU security is also reinforced as Greece seeks to further integrate European policies and already participates in the protection of EU’s external borders by supporting the Common European Maritime Policy and by participating in the reform of the procedures for the European Migration policy.

#### Hungary (2012)

Hungarian security threats link migration with drug trafficking, but migration is explored autonomously as it can carry public and national security risks. Though Hungary is mostly a transit country, it cannot be ruled out that an increasing number of illegal migrants will consider Hungary as a host country. The strategy focus on: (i) the cooperation with the EU in the Schengen Area and for external border protection; (ii) avoiding the marginalisation of migrants through effectively combating illegal migration and with the development of an integration strategy; (iii) countering illegal migration with the intensification of the fight against organised crime and human trafficking, as well as the further development of expulsion, repatriation and reintegration policies; (iv) an effective visa policy, and closer cooperation with third countries where migrants come from, with special emphasis on identifying and managing people posing a national security risk; and (v) the efficiency of procedures related to entry and residence permits as serious security risks can also be associated with legal migration.

#### Ireland (2015)

Conflicts as the ones in North Africa and the Middle East can lead to humanitarian crisis, resulting in increases in refugees, migrants and internally displaced people, and that is an overarching trend in the world today. As it is climate change that can influence population growth and migration flows. Or as it is smuggling of migrants and human trafficking as part of transnational organized crime, one the main security threats. Migration is explored as one overarching trend. Inward migration to the EU arises for various reasons including as a response to political conflict, environmental or economic pressures, potentially raising new challenges for host countries and for the EU.

#### Italy (2015)

Migration issues are mostly explored per feedback effects as demographic changes, scarcity of natural resources, conflict or poverty can originate increasing migratory pressure. The cooperation with the EU is important, especially about the security of the Euro-Mediterranean region as the Mediterranean basin has been part of the routes for illegal immigration, especially from the Horn of Africa and the Sahel.

#### Lithuania (2017)

One of the basic assumptions is that national security directly and indirectly depends on long-term challenges in Europe, being the management of irregular migration one of them, though it is a consequence of regional and global instability. One of the objectives is to support the EU migration and asylum policy by taking care that the migration and refugee crises caused by regional conflicts and other factors would not undermine the EU unity and stability. For this, ensuring credible protection of the EU external border and strengthening prevention and control of illegal migration is essential, as it is constant readiness to temporary reintroduce control at the EU internal border.

Low fertility rates, demographic ageing and large-scale emigration can pose a threat to Lithuania’s long-term social, economic and political stability and economic development. So, it is necessary to stimulate the return migration by ensuring the maintenance of political, economic, civic and cultural ties with the Lithuanians living abroad. Otherwise the economic growth and welfare sustainability can be compromised in the future.

#### Luxembourg (2015)

In the 2015 “Déclaration de politique européenne et étrangère”, migration issues are addressed in different domains. Firstly, by considering the migratory crisis as a source of instability for the EU, which should foster common and legal ways to promote the necessary immigration policy instead of feeding narratives that foster negative perceptions about migration. There is also a need for greater dialogue between EU and African countries, as poverty, repression, corruption and war in these countries fuel the flow of immigrants to the EU. Concerning the refugee crisis, Luxembourg shows its readiness to cooperate with EU by helping to relocate and to resettle refugees, and by offering human resources to Frontex and EASO.

#### Netherlands (2013)

Netherlands Defence Doctrine recognizes demographic changes as one of the major trends for the national and international environment. Migration can come from several drivers as food and water shortages, poverty, starvation and disease, more common in the developing world. Increasing migration and urbanisation can lead to ethnic tensions or increasing pressure on employment opportunities and social security systems, which could result in security risks. Also, the expected development of megacities will be accompanied by inevitable challenges in terms of infrastructure, public services, the environment and criminality, and will place enormous pressure on the quality of local government.

#### Poland (2013)

Migration is presented as a sector of national security, being necessary to prevent uncontrollable mass migrations of population within the territory of Poland. Feedback effects are explored, linking migration with climate change, armed conflicts, international organized crime and corruption, or demographic change. The rise of xenophobia and anti-immigrant attitudes within extreme-right is also pointed out when addressing the impacts of terrorism and political extremism.

The White Book also contains reference to Polish responsibilities to the EU and the Schengen area, existing a joint European strategy for EU protection of external borders and migration control with respect to combating illegal migration. The commitment with European legal frameworks for foreigners led to respect the entry of foreigners in the territory, their transit through the territory, residence in the territory, and leaving of the territory, as well as granting foreigners the refugee status, asylum, permit for tolerated stay, and temporary protection. Besides immigration policies, another important element of the state security should be the significant decreasing of people emigrating from Poland, restricting the process of economic migration among Polish citizens, particularly those who are young and well-educated.

#### Portugal (2013)

The impact of failed states and civil wars in refugees’ flows are taken as part of a risk to global security. The effects of climate change and transnational crime on human migration are also considered. Population ageing is understood as national challenge and the suggested way to cope with it is to promote new immigration policies, reinforcing integrating policies to avoid the rise of extreme-right, xenophobia and nationalism movements that can compromise social cohesion.

#### Slovakia (2013)

The Security Environment of the Slovak Republic identifies mass migration as an asymmetric threat that is expected to boost as failing states will increase. This may result in an uncontrolled migration to other states, an increase of intolerance and xenophobia towards immigrants, and manifestations of political extremism and conflicts based on cultural, religious, ethnic and lifestyle differences. Also, one of the main consequences of the political instability and recent developments in North Africa and the Middle East, among other regions, is the possible negative consequences on mass illegal migration.

#### Slovenia (2010)

Climate change, crisis areas, poverty and other social problems, terrorism and organised crime are global and transnational sources of threat and risk to national security and one of their indirect outcomes is an increase on migration flows, that can favour illegal migration and the trafficking of human beings. Illegal migration affects the Republic of Slovenia primarily because of routes running across its territory, particularly those from the area of South Eastern Europe, and is determined by the socio-economic and political-security situation in some regions. A greater scope of illegal migration may pose a general threat to the security and health of Slovenian nationals because the likelihood of the occurrence and spread of infectious diseases is additionally increased by, among other factors, migration. Epidemics and pandemics of infectious diseases are understood as potential medical and epidemiological threats.

The commitment to Schengen is also mentioned as the protection of EU external borders is one national security responsibility, especially in controlling and preventing illegal migration, avoiding illegal residence of immigrants in the country, and exchanging operational data with police forces of neighbouring countries, MS and the EU.

#### Spain (2013)

Irregular migratory flows are described as one of the main risks and threats that affect national security. In the Mediterranean, Spain, together with the EU and the international community, will back the efforts made by the countries to achieve better social and economic development and political stability. The regulation and the control of migratory flows are essential in the Maghreb. To prevent, control and manage migratory flows across borders, which also constitute outer limits of the EU, is a major goal in what concerns the management of migratory flows. That should occur according to EU System of Integrated Border Management framework and with cooperation of both origin and transit countries.

Feedback effects of irregular migration on organized crime and on terrorism with the possible radicalisation of both first- and second-generation emigrants settled in Spain are explored. Also, if poverty, inequality, wars, environmental risks, institutional weakness and authoritarian regimes persist in some countries, irregular migratory flows are expected to continue or increase in the future. As it may happen with other European countries, greater inadaptability can weaken social cohesion, increased exclusion can lead to social unrest, the emergence of urban ghettoes affects the integration of immigrants, and greater vulnerability of migrants can accentuate labour exploitation, human trafficking, or drug trade. So, it is important to defend the legality, to combat discrimination and to promote social integration and peaceful coexistence between immigrants and national citizens.

#### United Kingdom (2015)

Migration issues are part of both domestic and global security challenges, whether directly or due to the impact of instability like the conflicts in Syria and Iraq let to increasing mass migration and human trafficking. Organised immigration crime is one of the most serious and needs to be securitized. Also, climate change and resource scarcity can pose greater migration pressures. Migration is recognized as a global challenge. Instability, extremism and conflict in the Middle East and Africa have displaced millions of people in recent years. Many travelled to Europe, creating a humanitarian challenge and pressures across the EU. There is a need of coordinated work with multilateral agencies and countries that are hosting large numbers of refugees, to help improve livelihoods and give displaced people the best possible prospects as close to home as possible. It is also vital further strengthen on the ability to control migration, to offer protection to those who need it, and to ensure that the border, immigration and citizenship system can manage migration overseas, at the UK border and within the UK.

The UK is not part of Schengen open borders agreement, and so the approach to the migration crisis can be by taking refugees directly. The work with NATO and the EU will continue, as well as bilaterally, to deal with many subjects, being migration challenges one of them. All in all, there is a comprehensive approach to migration that ensures investment in countries of origin to help to reduce forced displacement and migration over the long term, humanitarian aid to those who are forcibly displaced, education and livelihood opportunities. Tackling the capacity of source and transit countries to manage their borders more effectively and organised immigration crime are priorities.

### A framework for analysis

Table 2 intends to summarise the presence of migration setting a framework for analysis that takes into consideration six dimensions derived from the migration-security theoretical review above-mentioned, namely: (i) presence of migration issues when characterising the global security environment; (ii) immigration issues considered as a risk to national security; (iii) security of (im)migrants; (iv) migration feedback effects with other risks and threats; (v) emigration as risk to social and economic security; and (vi) need for cooperation with the EU in migration policies and border control. For each dimension, a dual scale was used, in which (+) corresponds to a high exploration and (−) to a low or non-existent exploration. More in detail, in (i) and (ii) it was considered highly explored if migration, as a concept, was clearly presented and mentioned autonomously. For migrants’ security, it was considered highly explored if concerns with the security of (im)migrants was clearly expressed or even implied for more than one time, whether by exploring host countries’ integration policies in health, labour and socioeconomic issues or by recognizing the need of granting safe and open routes for people migrating. The feedback effects were considered highly explored if the documents elaborated at least two other issues that may be perceived as being connected to migration: climate change, population ageing, poverty, political and social instability, terrorism or transnational organised crime (especially human trafficking). If emigration flows were perceived as negatively impacting on national economic security, emigration was considered highly explored. Lastly, EU cooperation was considered highly explored if the cooperation with the EU and among other MS on both migration and asylum issues and border management was addressed within the documents.

**Table 2:** Migration issues in national security and defence strategies

Country	Global security trend	National security risk	Migrants’ security	Feedback effects	Emigration	EU cooperation
Austria	–	–	–	–	–	–
Belgium	–	+	–	–	–	–
Bulgaria	–	–	–	–	–	–
Croatia	–	–	–	–	–	–
Czech Republic	+	+	–	+	–	+
Denmark	–	–	–	–	–	–
Estonia	–	–	–	–	–	–
Finland	–	+	–	+	–	+
France	–	–	–	–	–	–
Germany	+	+	–	+	–	+
Greece	+	+	–	+	–	+
Hungary	–	+	–	+	–	+
Ireland	+	–	–	+	–	–
Italy	–	–	–	+	–	–
Latvia	–	–	–	–	–	–
Lithuania	–	–	–	+	+	+
Luxembourg	+	–	–	+	–	+
Malta	–	–	–	–	–	–
Netherlands	+	–	–	+	–	–
Poland	–	–	–	+	+	+
Portugal	–	–	–	+	–	–
Romania	–	–	–	+	–	–
Slovakia	+	–	–	+	–	–
Slovenia	–	+	–	+	–	+
Spain	+	+	–	+	–	+
Sweden	–	–	–	–	–	–
United Kingdom	+	+	–	+	–	–

This analysis reveals a divisive approach to migration-security nexus since there is a heterogeneous presence of migration issues in national security and defence strategies. The common structure lies especially on the exploration of feedback effects. Czech, German, Hungarian, Irish, Slovenian, Spanish and British documents are the ones conceptualizing migration issues autonomously instead of just linking them with other risks and threats. Emigration issues are almost forgotten for every MS, except for Lithuania and Poland. So, we may conclude that the securitization of migration is very much the securitization of immigration and is more focused on securing the nation-state and its population than securing the (im)migrants. Though migration is recognized as a transnational phenomenon that requires cooperation, there is an uncertain path on how MS address, and in some cases even ignore, the cooperation with the EU on migration issues besides integrated border management. In general, the lack of clear similar patterns of change reveals a divergent approach to migration-security nexus probably due to different security and defence strategic cultures inside the EU, though it is difficult to assess that just by analysing national security and defence strategies.

## The EU vision: Between fragmentation and integration

Though migration law and policies depend on MS, the EU has been playing an increasing role, especially after the Schengen Agreement and the Treaty of Amsterdam, which granted new competences to the EU regarding border control, bilateral agreements, visa system and asylum (see Arcarazo & Geddes, 2013). Cooperation in migration issues is not a general standardized process controlled by the EU, rather showing a bilateral, multilateral or intergovernmental nature, taking place between MS or between the EU and some MS. Like monetary policy, migration issues are also based on a partial integration that creates difficulties for both European institutions and MS, stressing the democratic deficit of EU institutions and establishing the need for interstate cooperation to construct common migration policies (Geddes, 1995). In the aftermath of the Schengen Agreement and the Dublin Convention, the EU began to reflect on the connection between immigration, terrorism, international crime and border control (Huysmans, 1995, p. 53). Border control must comply not only with the interests of MS, but also with the interests of the EU, and it seems clear that the political instability in the Mediterranean requires rethinking border management in that area. However, as the EU tends to put the humanitarian aspect of the migration crisis on the agenda, some national governments of MS tend to focus only on the security issues (Völkel, 2017, p. 83). With the deepening external dimension of EU asylum policies comes increasing complexity and interdependence, demanding multilevel governance which may benefit from the participation of both countries of origin and transit countries (Lavenex, 2016, p. 567). Though ‘EU’ropean engagement in the Mediterranean had become heterogeneous, where there are conscious diversified strategies, the lack of focus in this multilevel approach also helps to explain the range of unintended consequences on migration control in the Mediterranean region (Collyer, 2016, p. 621).

The difficulty to establish long-term agreements among MS regarding common migration and asylum policies seems evident, despite the progresses made since the first multiannual programme for Justice and Home Affairs. From Tampere (1999) to Stockholm (2010), with The Hague (2005) in between, the first decade of common policy in migration issues focused more on creating a baseline policy, dealing with border control and irregular immigration (Collett, 2014, p. 2). Though recent high inflows of asylum-seekers and refugees has brought to light the diversity of national approaches towards asylum and the issues of cooperation inside the CEAS, this is not news, since it has been a major issue of ‘EU’ropean asylum policy for the last two decades (Zaun, 2017, p. 241). EU lack of centralisation and leadership in managing immigration and asylum issues has ultimately led programmes to heavily depend on the cooperation between MS, resulting in hardly harmonised migration and asylum policies based on control and restrictive ideas. Thus, it is not yet clear how to address current issues such as the ISIS and new terrorist threats, the Lampedusa disaster and the management of the Mediterranean, and the so-called refugee crisis, among other challenges (Léonard & Kaunert, 2016, pp. 143–144). The European Agenda on Migration 2015 seemed to fill the after-Stockholm’s emptiness, identifying four core areas that need immediate action: reducing the incentives for irregular migration, strong asylum policy, saving lives and securing the external borders and a new policy on legal migration.

Since 2015, the migration crisis has gone to the centre of both European and national political agendas in a time when MS were still unequally and slowly recovering their economic growth and employment rates. The Mediterranean is a critical area in the transnational circulation of people, since it is the crossroads of different routes starting from Sub-Saharan Africa, the Middle East and Southwest Asia, which strengthen different South-North and South-South movements emerging from global demographic and economic unbalances (Ferreira, 2016b, p. 88). In the recent past, the Mediterranean was already at the centre of the European political agenda due to the Arab Springs. At that time, former French president, Nicolas Sarkozy, and the Italian president, Silvio Berlusconi, anticipated a migrant invasion that would require control and would even challenge the Schengen Agreement (Koff & Giraldo, 2015, p. 237). Migration crisis associated with international terrorism has resulted in the sudden closing of borders, as happened recently in France, the development of episodes of xenophobia and increasing populist anti-immigration narratives, revealing the incapacity of the EU and MS to create the space of tolerance and cultural diversity ambitioned in the nineties (Fulbrook & Cesarini, 1996, p. 217). Instead of centring the discussion on human rights, 2015 migration crisis debates were resting on burden-sharing and cross-Mediterranean cooperation (Klug, 2014, p. 51), also putting in evidence the absence of a collective memory of the European forced migration flows before and during the WW II (Fauri, 2015). The need for better cooperation does not imply that all MS must become hosting countries. It rather means that every MS should participate in a common strategy, either by hosting immigrants and refugees, or by sparing financial, human, and structural resources. However, there are several contradictory approaches, especially between transit countries such as Italy and Greece, main hosting countries such as Germany, France and Sweden, and countries belonging to the Visegrad group, for example (Ferreira, 2016b, pp. 91–93). Outside the EU, the Syrian conflict and political instability in MENA region do not seem to suggest a decrease in migration and refugee flows during the next years, which makes even more urgent to review the Dublin system (Ferreira, 2016a, p. 2). Restrictive policies will not stop irregular migration flows in the EU and will not increase internal security in ‘EU’rope (Völkel, 2017, p. 93). So, besides border control, agreements with origin and transit countries, it is also important to create safe routes and easier legal integration options, especially to labour migrants. About asylum-seekers it is needed to speed up the procedures and the coordination between the EU and MS. Lastly, when it concerns refugees, ‘EU’rope must rely on human rights to create a strategy for relocation and burden-sharing.

### EU Global Strategy

The EUGS is a key instrument to understand EU foreign and security policy and current view on the security challenges posed by migration issues since the EU is still waiting for a white paper on the future of ‘EU’ropean security and defence. It was approved in June 2016, and followed the European Security Agenda created by the Europe Council in June 2015. EUGS replaced the 2003 European Security Strategy (ESS) and the 2008 Report on the Implementation of the ESS. Far from intending a general analysis of the document, it seems to search for conceptual coordination between the Common Foreign and Security Policy (CFSP) and the Common Security and Defence Policy (CSDP) to cope with a new environment that must deal with previous Obama’s foreign policy retrenchment strategy, the Trump administration and the Brexit.

As far as migration and asylum are concerned in the EUGS (2016), the CSDP can be articulated with coastguard and border policing directed at ensuring the elimination of networks that traffic illegal migrants for profit. By assuming the current inadequacy of the EU migration and asylum policies, the need for a better understanding of migration-security nexus is evident. One of the priorities for external action relates to the state and social resilience to the South and East. Therefore, migration policy deserves a prominent role and requires special action in the countries of origin and transit of migrants and refugees. This action seems to favour mobility, legal migration, border management, readmission and return, by developing common actions between countries of origin and countries of transit, aimed to respond and prevent the deep causes of displacements, manage migration dynamics in receiving countries more efficiently, and fight transnational criminality. Regular channels for legal and circular human mobility must be guaranteed to block irregular flows, and this demands a common, more efficient European asylum system that may ensure a safe, regulated and legal arrival of refugees searching for international protection in the EU. By recognizing the transnational nature of migration, the approach demands the need to incorporate other international partners to guarantee the sharing of responsibilities and solidarity on a global level, especially with Turkey, one of the main transit countries outside the EU (European Union, 2016).

Two other issues seem relevant to point out: (i) the role of the EU in the future of currently highly unstable regions, such as North Africa, Sub-Saharan Africa and the Middle East, focusing its action on conflict resolution and promotion of human rights; and (ii) the development of multilateral relations involving political dialogue and cooperation in Central and Southern Asia and with different regional groups, in different domains including migration issues. In sum, a greater cohesion is needed in all foreign and internal policies concerning migration, as well as the definition of balanced policies respectful of the current framework of human rights of the European legal system, and the management of both flows and structural causes. This reality will only be possible if the present fragmentation of foreign policies relevant to migration and asylum issues is addressed, and that includes MS collaboration.

## Conclusion

Recent migration crisis has been difficult to tackle, and its future seems to depend on two critical uncertainties: the evolution of political and social instability in MENA region, and the future of the EU. In a time of globalisation, a blurring in the notions of border and a push towards a transformation in the concepts of citizenship and sovereignty could be expected, perhaps even threatening the Westphalian nation-state. However, globalization of migration led to increasing irregular migration flows and has resulted in the reinforcement of border control, has awaken growing nationalist narratives, and strengthened the separation between the national and the other. These tensions between global and national levels show that globalization is still an ongoing process with many resistance forces.

Migration and asylum policies within the EU depend on how the EU deals with globalisation and avoids new waves of protectionism among MS, sidestepping conflicted interests. These challenges test EU’s responses on controlling both internal and external borders, solving a humanitarian crisis, and managing asylum policies. To create a common migration and asylum system, there is a need for further reflection between national and European institutions on: (i) the EU’s role in a new world order, and how it affects the Atlantic partnership and the European Neighbourhood Policy (ENP); (ii) how essential can immigrants be for national economies and to sustain existing welfare systems in a context of population ageing and declining; (iii) how to avoid growing nationalism and extreme-right populism within MS’ political systems; and (iv) the acceptance of a multicultural EU with ethnic, religious and cultural differences. By assuming EU integration is still dealing with intergovernmentalism versus supranationalism, it is understandable the post Stockholm uncertainties and the looming future of the EU’s asylum policies as the CEAS presented several insufficiencies after the 2015 migration crisis. That led space to a ‘EU’ropean asylum policy dependent on a disjointed and de-centralised cooperation among MS. Though 2016 EUGS can be a standardising instrument for future national strategic revisions, both the EU and MS need higher strategic harmonisation to cope with the present migration crisis and to move towards a path of an effective common migration and asylum system.

This article enlightens different levels of securitization of migration among MS and a heterogeneous presence of migration issues in national security and defence strategies, showing different approaches to migration-security nexus inside the EU. By analysing both the national and the ‘EU’ropean levels, this article points out the EU has considered the humanitarian dimension of the migration crisis and the difficulties in the management of asylum-seekers and refugees procedures more strongly than MS, many times only focusing on the security dimension. When addressing the securitization of migration, the commitment to human security is insufficient if compared to national security on migration issues. Therefore, the securitization of migration seems to be much more directed to the nation-state rather than to people (immigrants, asylum-seekers and refugees). So far, when it comes to migration-security nexus, the institutional debate has been ruled by a national security lens, many times ignoring human insecurity of the people facing persecution in the country of origin or discrimination in their new country or even dying in transit. But if the protection of human rights and human security is excluded from national security concerns, in the long-run, new and more intense risks and threats may arise to compromise national security.

## Abbreviations

AIDS: acquired immune deficiency syndrome
CEAS: Common European Asylum System
CFSP: Common Foreign and Security Policy
CSDP: Common Security and Defence Policy
EASO: The European Asylum Support Office
ENP: European Neighbourhood Policy
ESS: European Security Strategy
EU: European Union
EUGS: European Union Global Strategy
FRONTEX: European Agency for the Management of Operational Cooperation at the External Borders of the Member States
HIV: The human immunodeficiency virus
ISIS: The Islamic State of Iraq and Syria
MENA: Middle East and North Africa
MS: Member States
NATO: The North Atlantic Treaty Organization
UK: United Kingdom
UN: United Nations
USA: United States of America
